# Role of Cytokines in Oligometastatic Non-Small-Cell Lung Cancer Treated with Stereotactic Radiation Therapy: An Observational Pilot Study

**DOI:** 10.3390/biom16030423

**Published:** 2026-03-13

**Authors:** Giorgio Facheris, Alessio Bruni, Valerio Nardone, Andrea Emanuele Guerini, Lorenzo Granello, Anna Gogna, Luca Triggiani, Michela Buglione di Monale e Bastia, Elisa D’Angelo, Stefania Bettelli, Francesca Di Pressa, Antonella Colosini, Giorgio Biasiotto, Roberto Bresciani, Paolo Borghetti

**Affiliations:** 1 Department of Radiation Oncology, ASST Spedali Civili and University of Brescia, 25100 Brescia, Italy; giorgio.facheris@gmail.com (G.F.); a.e.guerini@gmail.com (A.E.G.); luca.triggiani@unibs.it (L.T.); michela.buglione@unibs.it (M.B.d.M.e.B.); paolobor82@yahoo.it (P.B.); 2 Radiotherapy Unit, Department of Oncology and Hematology, Azienda Ospedaliero-Universitaria di Modena, 41100 Modena, Italy; bruni.alessio@aou.mo.it; 3 Department of Medical and Surgical Sciences, University of Modena and Reggio Emilia, 41100 Modena, Italy; 4 Department of Precision Medicine, University of Campania “l. Vanvitelli”, 80100 Naples, Italy; v.nardone@hotmail.it; 5 Department of Molecular and Translational Medicine, University of Brescia, 25100 Brescia, Italy; anna.gogna@unibs.it (A.G.); anto.colo90@libero.it (A.C.); roberto.bresciani@unibs.it (R.B.); 6 Radiation Oncology Department, Bellaria Hospital, AUSL of Bologna, 40100 Bologna, Italy; elisa.dangelo@ausl.bologna.it; 7 Unit of Molecular Pathology and Predictive Medicine, University Hospital of Modena, 41100 Modena, Italy; bettelli.stefania@aou.mo.it; 8 Department of Radiation Oncology, Alessandro Manzoni Hospital, 23900 Lecco, Italy; francesca.dipressa@gmail.com; 9 Highly Specialized Laboratory, ASST Spedali Civili di Brescia, 25100 Brescia, Italy

**Keywords:** cytokines, SBRT, NSCLC

## Abstract

Introduction: Stereotactic radiotherapy (SRT) is increasingly used in oligometastatic non-small-cell lung cancer (NSCLC) and is known to elicit systemic immune effects, although the underlying mechanisms remain not fully understood. Methods: In this prospective pilot study, we evaluated plasma cytokine variations in 19 patients with oligometastatic or oligoprogressive NSCLC undergoing SRT. Peripheral blood samples were collected before treatment (T0) and one month after SRT (T1) and the concentrations of nine cytokines (IFN-γ, IL-1β, IL-2, IL-4, IL-6, IL-10, IL-12p70, IL-17A and TNF-α) were quantified using a multiplex Luminex assay. Non-parametric tests and Cox regression models were used to investigate associations between cytokine levels, clinical variables, systemic treatments, and survival outcomes. SRT induced significant post-treatment increases in IFN-γ, IL-2, and IL-6, consistent with systemic pro-inflammatory activation and T-cell stimulation. Cytokine dynamics were influenced by patient- and tumor-related factors: female sex was associated with higher IL-2 and TNF-α levels; oncogene-addicted tumors showed lower IL-6 levels; and oligoprogressive disease exhibited attenuated cytokine variations compared with metachronous oligometastatic disease. Tyrosine kinase inhibitors were associated with globally reduced cytokine levels and blunted IL-1/IL-2 changes, whereas patients receiving immune checkpoint inhibitors displayed higher IL-2 and IL-6 concentrations and greater post-SRT increases in IFN-γ. Oncogene-addicted status and IL-12 variation emerged as independent predictors of overall survival and a composite model integrating these variables significantly stratified prognosis. Conclusions: These findings suggest that SRT triggers measurable systemic immune activation in oligometastatic NSCLC, which is further shaped by tumor biology, disease burden, and concomitant systemic therapies. Although limited by the small sample size, this study supports the feasibility and potential utility of cytokine profiling to refine patient selection and guide biomarker-driven combinations of SRT with targeted and immune-based treatments, warranting validation in larger prospective cohorts.

## 1. Introduction

In recent years, the treatment of non-small-cell lung cancer (NSCLC) has significantly evolved due to the advancements in immunotherapy and target therapy.

The therapeutic landscape for non-resectable NSCLC has been redefined by the consolidation of immunotherapy after chemoradiotherapy, as established by the PACIFIC trial [1]. Furthermore, for patients harboring specific mutations, such as EGFR, the introduction of targeted therapies like osimertinib has further shifted the standard of care toward more personalized strategies [1]. However, the management of patients who develop oligometastatic or oligoprogressive disease remains a clinical challenge where the role of local treatments like stereotactic radiation therapy (SRT) is increasingly relevant.

Particularly, the impact of immune checkpoint inhibitors on patient prognosis seems to be relevant in both metastatic and locally advanced settings [2,3]. Concurrently, improvements in image guidance, dose delivery, and organ motion control have led to the development of stereotactic radiation therapy (SRT), which delivers high doses per fraction to the tumor while sparing surrounding healthy tissues. SRT has shown high clinical effectiveness in local control of both early and metastatic diseases. Although the underlying biological mechanisms behind SRT effects remain poorly characterized, they most likely involve vascular damage and immune system stimulation, leading to indirect tumor cell death [4,5]. The synergy between radiotherapy and the immune system is a complex, double-edged process. While radiation can promote a pro-inflammatory environment through the release of damage-associated molecular patterns (DAMPs) and the recruitment of effector T cells, it can also trigger immunosuppressive mechanisms that lead to treatment resistance [6]. This “cancer-immune cycle” is heavily influenced by the systemic cytokine milieu; soluble mediators not only reflect the local response to ionizing radiation but also the broader state of immune fitness or exhaustion [7]. Understanding these dynamics is crucial to optimize patient selection and the timing of combined treatment modalities.

SRT-induced tumor cell death releases antigens that may induce the expression of major histocompatibility complex class 1 (MHC-1), promoting antigen-presenting cell (APC) activity, priming lymphocytes, and inducing antitumor adaptive immunity. This could explain the “abscopal effect,” which happens when radiation causes regression of tumors outside the irradiated area [8]. Furthermore, cytokines and chemokines play a central role in coordinating the immune response, being involved in various aspects of tumor biology, including antitumoral immunity and carcinogenesis-promoting processes like chronic inflammation and immunotolerance [9,10,11].

The systemic immune landscape in NSCLC is orchestrated by a complex network of cytokines that modulate the balance between antitumor activity and immune evasion. In the context of radiotherapy, pro-inflammatory mediators such as IL-6, IL-12, and TNF-α are of particular interest, as they drive the initial response to radiation-induced cellular stress and can promote the recruitment of effector T cells. Conversely, the release of anti-inflammatory or immunosuppressive cytokines, including IL-10 and TGF-β, may counteract these effects, potentially leading to treatment resistance or immune exhaustion. Rather than a rigid Th1/Th2 classification, the dynamic shift in these soluble factors during SRT reflects the treatment’s capacity to reshape the host’s immune polarization. Therefore, monitoring these specific biomarkers provides a window into the systemic biological impact of SRT beyond local tumor control. Rather than a broad functional classification, the cytokines selected for this study were chosen based on their established roles in the NSCLC microenvironment and their sensitivity to ionizing radiation. For instance, pro-inflammatory mediators such as IL-6 and TNF-α are known to orchestrate the systemic inflammatory response to radiation-induced tissue damage, while the IL-12 family plays a pivotal role in bridging innate and adaptive post-radiotherapy immune responses. By monitoring these specific markers, we aim to capture the dynamic shift between immune activation and potential treatment-induced exhaustion [10]. Despite numerous clinical trials exploring the interaction between immunotherapy and radiotherapy, the exact physiological mechanisms remain largely unknown. Limited data are available regarding the temporal variations in plasma cytokine concentrations induced by radiotherapy in NSCLC. While some studies have explored these dynamics in early-stage disease, to our knowledge, there is a paucity of prospective evidence specifically focusing on the metastatic setting. The present work, therefore, aims to provide a preliminary analysis of inflammatory mediators in this specific cohort to identify potential radiation-induced patterns [10,11,12]. The present work aims to conduct a preliminary analysis of plasma concentration of several inflammatory mediators in NSCLC patients undergoing SRT, to identify radiation-induced secretion patterns and possible correlations with known pathophysiological mechanisms [13]. Finally, this study emphasizes the need to address feasibility and methodological issues to design a future, larger prospective study.

## 2. Materials and Methods

This prospective pilot study analyzed the plasma concentration of various inflammatory mediators in patients with oligometastatic NSCLC undergoing SRT. Patient recruitment was conducted between January 2020 and January 2023 in two different Italian radiation oncology department.

Patients were required to meet the following eligibility criteria: age ≥ 18 years; a diagnosis of oligometastatic or oligoprogressive NSCLC (defined as fewer than four metastases in a maximum of two organs); and all lesions eligible for SBRT. Specifically, oligometastatic disease was defined as the development of metastases during follow-up in a previously non-metastatic setting, whereas oligoprogressive disease referred to the progression of one or more lesions already known at the time of diagnosis. SBRT regimens consisted of 1 to 8 fractions, with a minimum dose of 6 Gy per fraction.

Finally, all patients provided written informed consent.

For each patient, 10 mL of peripheral blood was collected before SRT (T0), one month after (T1) and then every four months during the first year and every six months until disease progression. In a previous study, the comparison between concentrations before and after SRT was evaluated. However, measurements taken within the 3-to-7-day interval did not yield significant results. Based on these findings, in the present study, we decided to omit the immediate post-SRT measurement and instead retain the one-month follow-up to compare it with the baseline [13].

Plasma was separated by centrifugation and stored at −80 °C. The concentration of inflammatory mediators was measured using a multiplex immunoassay on the Luminex MAGPIX platform (Version 4.3). Measurements were performed in duplicate for each sample, and the averages were used to minimize stochastic error. Values not directly measurable were extrapolated from the analysis software; out-of-range values were approximated to the lowest detected value for that analyte, reduced by one decimal place. The study was conducted in accordance with the Declaration of Helsinki and the Harmonized ICH Guidelines for Good Clinical Practice, with approval from the local Ethics Committee of the participating centers, Comitato Etico di Brescia (approval code: NP 3553; approval date: 16 December 2019).

### 2.1. Blood Processing and Multiplex Analysis

Blood samples (10 mL) were drawn in EDTA-containing tubes and were centrifuged at 2000× *g* at 4 °C for 10 min within one hour after collection. The supernatant obtained was stored at −80 ° C until analysis. Serum levels of 9 cytokines (IFNγ, IL-1β, IL-2, IL-4, IL-6, IL-10, IL-12 p70, IL-17A and TNFα) were measured using a Cytokine/Chemokine/Growth Factor kit according to the manufacturer’s instructions (9-Plex Human Panel, High Sensitivity—Cat. No. EPXS090-12199-901). Briefly, a standard curve was prepared through serial dilution of antigen standards. The assay was performed on 96-well plates provided with the kit. Premixed magnetic microspheres conjugated to specific antibodies were added to each well and subsequently washed twice with diluted wash buffer. Standards and undiluted samples were added to the wells, shaken for 30 min at 500 rpm at room temperature, and then incubated overnight at 4 °C. At the end of the incubation, excess material was removed with two washes and 25 μL of detection biotinylated antibodies were added to the wells and shaken at 500 rpm for 30 min. Wells were washed 2 times and incubated with 50 μL of Streptavidin-Ficoerythrin with shaking at 500 rpm for 30 min. After further washing, 120 μL of Reading Buffer was added with shaking at 500 rpm for 5 min. Cytokine levels were measured using the Bio-Plex tool MAGPIX Multiplier Reader (BIO-RAD Laboratories, Hercules, CA, USA) and data analysis was performed using Luminex software (BIO-RAD Laboratories, Hercules, CA, USA)

### 2.2. Statistical Analysis

All statistical analyses were performed using SPSS Statistics software (version 24). A *p*-value of <0.05 was considered statistically significant. The differences in cytokine concentrations between baseline (T0) and post-treatment (T1) were assessed using the Wilcoxon signed-rank test to account for non-parametric distributions and small sample sizes. The correlation between the level of cytokines (at baseline, post-SABR, and their relative variation) and the categorical clinical variables (sex, age, oncogene addiction status, use of TKIs and use of immune checkpoint inhibitors) were analyzed using the chi-square test. Progression-free survival (PFS) and overall survival (OS) was analyzed using Cox proportional hazards regression. PFS is defined as the time from the start of SRT to disease progression or last follow-up without progression. OS is defined as the time from SRT to death from any cause or last follow-up. The impact of cytokine levels (at baseline, post-SRT, and delta variations) and clinical variables on survival outcomes were examined.

## 3. Results

### 3.1. Sample Characteristics and Cytokines Concentrations

A total of 31 patients were enrolled in the study, 22 at the Radiation Oncology Department of ASST Spedali Civili and University of Brescia and 9 at the Radiation Oncology Department of the University Hospital of Modena. The cytokine assay was performed only on 19 of these patients, as it was not possible to obtain the sample one month after the end of SRT for the remaining subjects due to intercurrent causes (COVID-19 pandemic, no-show at follow-up visit, death from other causes, or failure to administer radiotherapy treatment). The descriptive analysis of the population thus refers only to the patients for whom biochemical and clinical data were available.

The descriptive data collected, such as disease characteristics including histology, disease stage (oligoprogressive or oligometastatic), presence of driver mutations, previous systemic therapies, number of lesions treated and dose of SRT, are examined and described in Table 1.

The patient cohort consisted of 13 women (68.4%) and 6 men (31.6%). The median age was 68 years. The most prevalent histology observed was adenocarcinoma (16 patients; 84.2% of cases), while 2 patients (10.5%) had squamous cell carcinoma and only 1 patient had sarcomatoid carcinoma (5.3%).

Analysis of target mutations revealed EGFR mutation in five patients (26.3%), ALK mutation in one (5.3%), RET mutation in two (10.5%), BRAF V600E mutation in one (5.3%) and KRAS mutation in four (21.1%). No subjects showed mutations in the RET, ROS1, or NTRK genes.

PD-L1 expression was <1% in four patients (21.1%), between 1% and 50% in eight (42.1%) and over 50% in seven (36.8%).

Eight participants (42.1%) were metachronous oligometastatic and 11 (57.9%) were oligoprogressive; no synchronous oligometastatic cases were recruited in the analyzed.

Regarding systemic treatments prior to SRT, 10 patients (52.6%) had undergone chemotherapy (9 with one line, 1 with two lines), 4 patients (21.1%) had received first-line tyrosine kinase inhibitors (TKIs) and 7 (36.8%) had received first-line immunotherapy. No patients were submitted to SRT while receiving chemotherapy, while six (31.6%) were receiving immunotherapy and another six were receiving TKIs.

SRT was delivered to brain lesions in five cases (26.3%) and to lung sites in seven (36.8%); bone metastases accounted for 10.5% of total cases (three patients), while adrenal or liver metastases were treated in two patients (10.5%) respectively. Just in one case (5.3%), two sites—bone and adrenal gland—were treated simultaneously.

The fractionation regimens used were: 21–24 Gy in single fraction for brain lesions; 50–55 Gy in five fractions for lung lesions; 27–30 Gy in three fractions or 40 Gy in eight fractions for bone targets; 60 Gy in three fractions or 50 Gy in five fractions for liver metastasis; and 40 Gy in eight fractions or 54 Gy in nine fractions for metastases of the adrenal gland.

Mean time between T0 and T1 sample collection was 36 days, with a median of 33 days. During follow-up, 15 patients (78.9%) experienced disease progression, 11 of whom remained oligometastatic, while 4 widely progressed.

At time of data analysis (September 2023), 12 patients (63.2%) were still alive, while six (31.6%) had died due to disease progression and one (5.3%) from non-cancer-related causes.

### 3.2. Comparison of Cytokine Levels Pre- and Post-SRT

**Figure 1:**
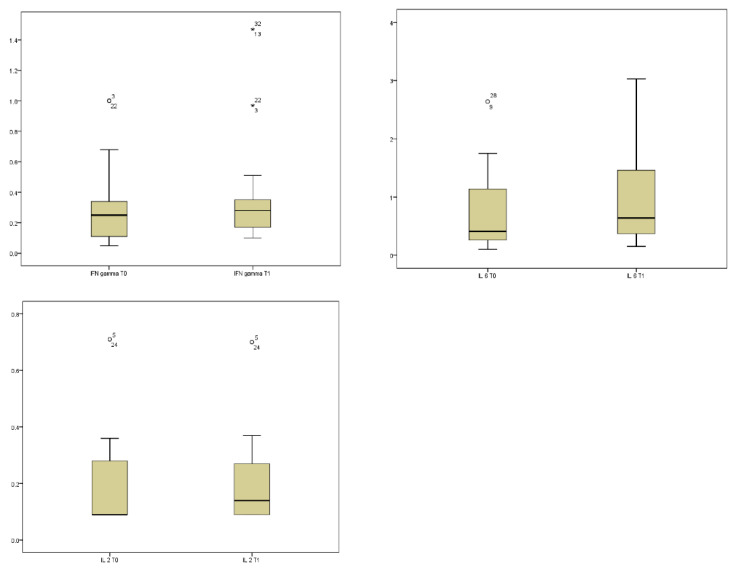
Longitudinal variations in plasma cytokine levels following stereotactic radiation therapy (SRT). Boxplots represent the distribution of IFN-gamma, IL-6 and IL-2 concentrations (pg/mL) measured at baseline (T0) and at one month after SRT (T1) in our sample. Statistical significance was determined using the Wilcoxon signed-rank test.

The comparison of cytokine concentrations between baseline (T0) and one month post-SRT (T1) revealed statistically significant changes in specific pro-inflammatory mediators. The sign test identified significant increases in IFN-γ (*p* = 0.044, mean T0 0.28 ± 0.22, mean T1 0.36 ± 0.32), IL-2 (*p* = 0.038, mean T0 0.17 ± 0.15, mean T1 0.19 ± 0.14), and IL-6 (*p* = 0.048, mean T0 0.74 ± 0.65, mean T1 0.96 ± 0.79) following treatment (Figure 1).

### 3.3. Association Between Clinical Variables and Cytokine Levels

Using chi-square analysis, several significant associations were identified between clinical variables and cytokine levels (both at baseline and in terms of post-treatment variation):

- Gender: Statistically significant differences were observed in the expression of IL-2.T1 (*p* = 0.011, mean in males 0.18 ± 0.12, mean in females 0.23 ± 0.09), and TNF-α.T1 (*p* = 0.021, mean in males 0.09 ± 0.07, mean in females 0.18 ± 0.15), resulting higher in females, suggesting a gender-specific immune response profile in patients undergoing SRT.
- Age: No significant correlations were found between age and baseline cytokine levels or their variation over time.
- Oncogene addiction status: Patients with oncogene-addicted tumors exhibited lower levels of IL-6 at both timepoints (*p* = 0.003, mean in oncogene-addicted 0.48 ± 0.35 at T0 and 0.60 ± 0.41 at T1 versus mean in non-oncogene-addicted 1.09 ± 0.80 at T0 and 1.47 ± 0.91 at T1) and were more likely to be treated with TKIs (*p* = 0.031), implicating a link between molecular tumor subtype and systemic inflammatory activity.
- Disease burden (oligometastatic vs. oligoprogressive): Significant associations were found with delta variations in IL-2 (*p* = 0.024, mean delta in oligometastatic 0.58 ± 0.06 versus mean delta in oligoprogressive 0.001 ± 0.08), IL-4 (*p* = 0.033, mean delta in oligometastatic 0.09 ± 0.29 versus mean delta in oligoprogressive −0.09 ± 0.21), and TNF-α (*p* = 0.012, mean delta in oligometastatic 0.027 ± 0.12 versus mean delta in oligoprogressive −0.22 ± 0.11), indicating differential immune dynamics based on metastatic extent with lower levels for oligoprogressive disease.
- Use of TKIs (concurrent or previous): Treatment with TKIs was associated with reduced baseline levels of IL-1 (*p* = 0.031, mean in patients using TKIs 0.10 ± 0.044 versus mean in patients not using TKIs 0.23 ±0.18), IL-2 (*p* = 0.030, mean in patients using TKIs 0.09 ± 0.01 versus mean in patients not using TKIs 0.20 ±0.18), IL-6 (*p* = 0.022, mean in patients using TKIs 0.38 ±0.34 versus mean in patients not using TKIs 0.90 ±0.70), and TNF-α (*p* = 0.007, mean in patients using TKIs 0.037 ± 0,02 versus mean in patients not using TKIs 0.20 ±0.20), as well as significant delta variations in IL-1 (*p* = 0.017, mean in patients using TKIs 0.046 ± 0.06 versus mean in patients not using TKIs −0.01 ±0.05) and IL-2 (*p* = 0.003, mean in patients using TKIs 0.09 ± 0.07 versus mean in patients not using TKIs −0.05 ±0.06).
- Use of immune checkpoint inhibitors (ICIs) (concurrent or previous): Patients receiving ICIs had significantly baseline higher levels of IL-2 (*p* = 0.007, mean in ICI patients 0.27 ± 0.23 versus mean in patients not using ICIs 0.12 ± 0.08) and IL-6 (*p* = 0.019, mean in ICI patients 1.10 ± 0.79 versus mean in patients not using ICIs 0.57 ± 0.51), and exhibited significant post-treatment increases in IFN-γ (ΔIFN-γ, *p* = 0.010, mean in ICI patients −0.11 ± 0.14 versus mean in patients not using ICIs 0.12 ± 0.15) compared with patients who did not received ICIs, suggesting enhanced immunostimulation in this subgroup.

### 3.4. Survival Analysis

At a median follow-up of 14.3 months, the 5-year overall survival (OS) rate for the entire cohort was 70.1%, with a median OS of 73 months. The median progression-free survival (PFS) was 11 months. 

There was no correlation between any of the analyzed parameters and PFS.

**Figure 2:**
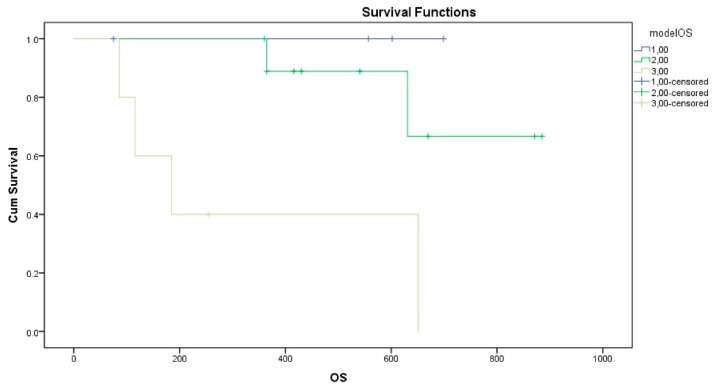
Kaplan–Meier survival analysis stratified by the proposed prognostic model. Patients with metastatic NSCLC were categorized into three risk groups based on their oncogenic driver status and IL-12 longitudinal dynamics. Group 1: Oncogene-addicted patients exhibiting a reduction in IL-12 levels between T0 and T1; Group 2: Oncogene-addicted patients with increased IL-12, or non-addicted patients with reduced IL-12; Group 3: Non-addicted patients exhibiting increased IL-12 levels.

For OS analysis, both oncogene-addicted status (*p* = 0.003, mean OS for oncogene-addicted 791 days ± 46 days versus mean OS for non-addicted 483 days ± 83 days) and the delta variation in IL-12 (*p* = 0.005, mean OS for patients with delta IL-12 < 798 days ± 55 days versus mean OS for patients with delta IL-12 ≥ 530 days ± 61 days) were significantly associated with OS. A composite prognostic model incorporating these two variables was developed (Group 1: oncogene-addicted and IL12 reduced; Group 2: oncogene-addicted and IL12 increased OR NOT oncogene-addicted and IL reduced; Group 3: NOT oncogene-addicted and IL12 increased) and demonstrated a significant stratification of survival outcomes (log-rank *p* = 0.01) (Figure 2). Mean OS for Group 1: 720 days ± 37 days (all cases are censored); mean OS for Group 2: 770 days ± 48 days; mean OS for Group 3: 337 days ± 87 days. This supports the potential utility of integrating molecular tumor characteristics and immune biomarker dynamics to improve prognostication in patients with oligometastatic NSCLC treated with SRT.

## 4. Discussion

The aim of this study was to identify biomarkers predictive of local and systemic response to radiotherapy by analyzing the plasma concentrations of multiple inflammatory mediators in patients with oligometastatic NSCLC undergoing SRT. Indeed, the variations in the concentrations of nine cytokines were examined, demonstrating the feasibility of using liquid biopsy for proteomic characterization in 19 patients with oligometastatic NSCLC undergoing SRT. Moreover, already known clinical factors impacting NSCLC prognosis were included in the analysis to develop predictive models.

These results show the known potential of SRT to induce a measurable systemic immunologic response, characterized by an alteration in key cytokines associated with both pro-inflammatory signaling and T-cell activation. Indeed, IFN-γ, IL-2, and IL-6 significantly increased following treatment (Figure 1).

Coherently, the administration of ICIs, which exert their effect by enhancing antitumor immune response, resulted in significantly higher levels of IL-2 and IL-6. Moreover, patients receiving ICIs exhibited significant post-treatment increases in IFN-γ, similarly to SRT.

Therefore, effects of SRT and ICIs could both stimulate antitumor response and result in mutual enhancement of antineoplastic activity.

In this study, statistically significant post-SRT increases in IFN-γ, IL-2, and IL-6 at one month were observed, indicating evidence of systemic immune activation. Similar patterns have been described in previous prospective SBRT studies in NSCLC, where peripheral immune profiling demonstrated induction of interferon-γ signaling, expansion of circulating proliferating (Ki-67^+^) CD8^+^ and CD4^+^ T-cell subsets, and enrichment of activated T-cell gene signatures following high-dose thoracic irradiation [14].

The increase in IL-6 observed post-SABR in our cohort is also consistent with published findings from several studies focused on thoracic RT. In multiple prospective analyses, IL-6—together with IL-8 and TGF-β1—emerged as one of the most frequently modulated cytokines during or after lung radiotherapy. Its increase was associated both with systemic inflammatory responses and major risk of radiation pneumonitis [15].

Changes in IFN-γ and IL-2 after RT have been reported across tumor sites and radiation modalities, although the kinetics and magnitude vary with dose, fractionation, and sampling time. In early-stage NSCLC, SBRT has been associated with transient increases in IFN-γ and IL-2 detectable within weeks of treatment, consistent with the adaptive immune activation profile seen in our data [16].

This subgroup analyses suggest potential modulatory effects of concomitant systemic therapies. Patients receiving ICIs demonstrated higher baseline IL-2 and IL-6, in line with clinical trial evidence that SBRT combined with PD-1/PD-L1 blockade can enhance systemic immune responses, “heat up” immunologically cold tumors, and improve objective response rates in metastatic NSCLC [6,17,18].

In contrast, patients treated with TKIs exhibited reduced levels of IL-1, IL-2, IL-6, and TNF-α, as well as smaller delta changes for IL-1 and IL-2. While oncogene-driven NSCLC is known to display a distinct tumor immune microenvironment and TKIs can alter systemic immunity [6,19,20], we found no studies directly reporting this cytokine suppression pattern. These findings may therefore represent a novel clinical observation warranting further validation.

Similarly, these data indicate higher IL-2 and TNF-α levels in female patients and different cytokine delta patterns in oligometastatic versus oligoprogressive disease. Although gender-related immune differences have been documented in oncology [21,22], to our knowledge no studies have reported this specific pattern, and the observed burden-related cytokine changes have limited precursors in the literature; the observed cytokine profiles in the oligoprogressive subgroup might be linked to a state of chronic antigen exposure, a condition known to drive T-cell dysfunction or exhaustion in advanced malignancies [23]. In these patients, the persistent tumor burden may lead to the sustained release of immunosuppressive factors, which in turn blunts the pro-inflammatory stimulus typically induced by radiotherapy. While our findings regarding this exhaustion-like phenotype are speculative, they align with current models of cancer immune evasion [24] and suggest that SRT alone may be insufficient to reinvigorate the immune response in the context of high systemic tumor load. Both observations should thus be interpreted as hypothesis-generating.

Finally, this study underscores the prognostic significance of integrating molecular oncogenic status and immune biomarker dynamics in predicting OS in patients with oligometastatic NSCLC treated with SRT. Specifically, we identified that both oncogene addiction status and changes in interleukin-12 (IL-12) levels were independently associated with OS, consistent with previous findings emphasizing the role of tumor biology and immune milieu in lung cancer outcomes.

The use of SRT during immunotherapy in stage IV NSCLC has been analyzed and is still under analysis in multiple clinical studies; for example, the COSINR study [25] is a phase I study that randomized 35 patients to receive a double therapy with concomitant or sequential ICI and SRT. Another trial is the phase II study driven by Welsh et al. [26] that randomized 72 patients to receive radiotherapy (stereotactic or normo-fractionated) + pembrolizumab compared to pembrolizumab in monotherapy. Finally, the phase II PEMBRO-RT study involved 76 patients and randomized them into two arms: sequential pembrolizumab after SRT on a single lesion (24 Gy in three fractions) compared to pembrolizumab in monotherapy. The ORR was 36% and 18%, respectively. Additionally, PFS favored the arm with SRT (6.6 months compared to 1.9 months), as did OS (15.6 months compared to 7.6 months) [20].

In this case series, the greatest variations were in the pro-inflammatory cytokine TNF-α and the anti-inflammatory cytokine IL-4, which showed an increase and a decrease, respectively, as expected after stereotactic stimulation in patients with ongoing immunotherapy.

The oligometastatic setting includes heterogeneous entities. As seen in our analysis, the metachronous and oligoprogressive subgroups behave differently on a biochemical and clinical level. In our case series, the behavior of the measured cytokine levels suggests that metachronous oligometastatic disease is more responsive at the tumor microenvironment level to the pro-inflammatory stimulus given by SRT [27]. Thus, cytokines with both pro-inflammatory and anti-inflammatory activity have shown an increase in their concentration, the former likely due to a direct effect of the actinic stimulus, the latter as a regulatory intervention of the inflammatory response itself. However, the same phenomenon was not observed in the oligometastatic scenario, while a reduction in anti-inflammatory cytokines was identified. This may suggest that the microenvironment in these patients is in a basal status of greater inflammatory stimulus. To explain this phenomenon, we may hypothesize that in the oligoprogressive disease, there is a metastatic set with a higher load representing a source of persistent exposure of tumor antigens to T cells, which gradually lose their effector functions due to exposure to inhibitory factors at the TCR level.

In the case of metachronous oligometastatic disease, however, the lower tumor burden that is treated in its entirety allows for a reduction in tumor load and therefore the exposure of immune cells to antigens. Consequently, the concept of the “reinvigoration-to-tumor-burden” ratio may be considered as predictive of a positive response to ICI therapy (if increased) [28].

While the aforementioned results are grounded in empirical data, the following interpretations regarding immune exhaustion and tumor burden are proposed as hypotheses to be further explored.

In this sample, the analyses of patients treated with TKIs for oncogene-addicted NSCLC showed a behavior of IL-1 opposite to that of wild-type disease, confirming the presence of a tendency to develop a more inflammatory microenvironment. Notably, a trend towards decrease in the immunosuppressive IL-17 was identified in the oncogene-addicted population. These results confirm the existence of a tumor microenvironment that, in the context of oncogene-addicted disease, tends to be more pro-inflammatory, with greater lymphocytic infiltration and higher concentration of cytokines activating the inflammatory pathway, especially when EGFR and ALK inhibitors are used [29].

The oncogene-addicted subgroup demonstrated a distinct survival advantage, aligning with established evidence that driver mutations such as EGFR, ALK, or ROS1 rearrangements predict responsiveness to targeted therapies and may influence radiosensitivity. Clinical data suggest that EGFR-mutant NSCLC cells are radiosensitive, supporting the role of precision radiotherapy based on EGFR mutation status in metastatic NSCLC. Additionally, combining radiotherapy with targeted therapies in NSCLC has shown improved efficacy, highlighting the importance of integrating molecular profiling into treatment strategies [30,31].

In parallel, the dynamic change in IL-12—a critical cytokine involved in antitumor immunity through activation of natural killer and T cells—emerged as an important immune biomarker. A reduction in IL-12 levels post-treatment was associated with poorer OS, potentially reflecting an impaired systemic immune response or tumor-induced immunosuppression. IL-12 has been shown to activate antitumor cytotoxic immune responses and inhibit immune suppression, suggesting its potential as a therapeutic agent in cancer immunotherapy [32].

The composite prognostic model integrating both oncogene-addicted status and IL-12 variation stratified patients into three groups with significantly different survival outcomes, suggesting additive or possibly synergistic effects between tumor-intrinsic genetic drivers and the host immune environment. This approach exemplifies precision medicine, wherein combining molecular and immune parameters can refine risk stratification beyond conventional clinical factors alone. Emerging evidence supports the integration of targeted therapies with radiotherapy in advanced NSCLC, underscoring the potential benefits of such combinatory approaches [31]. It should be noted that the data-derived prognostic model has no real clinical applicability, but it may be useful for hypothesis-generating studies aimed at combining clinical and biological variables to more accurately predict the benefit of SRT in oligometastatic disease. The role of concomitant or ongoing therapies, comorbidities, and multiple clinical confounding factors was not fully explored in this study, and this in itself constitutes a limitation, but at the same time, an interesting starting point for further research.

Although the results of this study are innovative and could be considered as hypothesis-generating for subsequent (prospective) studies, multiple limits must be acknowledged. First of all, the nature of this study is just exploratory, being based on a limited sample, as envisaged by the initial design of this pilot research. Furthermore, the onset of the SARS-CoV-2 pandemic further hindered patient enrolment. It could also explain some characteristics of our sample that differ from the typical NSCLC population. For example, our population is largely represented by oncogene-addicted patients and therefore characterized by better OS and PFS. This finding resulted in better survival rates compared to those reported in the literature for patients at this stage, so our results may not accurately match real-life data. As multiple cytokines play a dual and opposing action on immune response, interpretation of data from a small sample could be challenging.

Our experience focuses on the clinical unmet need to identify new biomarkers able to predict a benefit from stereotactic radiation therapy in the context of oligometastatic disease and to understand the hidden mechanisms underlying a potential systemic response to a local treatment. Furthermore, the results of this study showed how the integration of clinical and proteomic features into a predictive composite model could potentially be useful to stratify patients with oligometastatic NSCLC who can truly benefit from stereotactic radiotherapy.

Globally, our results must be considered with caution, but at the same time they can lay the foundation for a stronger rationale for larger and more targeted studies to identify the role of proteomic biomarkers as factors able to define subgroups of patients who benefit from ablative radiotherapy. Taken together, our findings advocate for future studies, investigating the role of proteomics in the management of oligometastatic NSCLC. It will be crucial to plan clinical trials with larger cohorts with the aim of prospectively validating this composite model and exploring its utility in guiding combination therapies, such as adding immunomodulatory agents.

It is important to note that the observed variations in cytokine profiles may be influenced by factors beyond the immediate effects of SRT. Alternative drivers, such as systemic inflammation secondary to tumor progression, the residual impact of prior therapies, or underlying patient comorbidities, could contribute to these changes. Given the preliminary nature of this evidence, these findings should be interpreted with caution, and their direct clinical applicability requires further validation in controlled settings.

To conclude, our findings confirm and extend prior evidence that SABR induces systemic immune modulation in NSCLC. The associations with systemic therapies, gender, and metastatic burden observed here require prospective confirmation in larger cohorts.

## 5. Conclusions

SRT was associated with significant systemic immunologic changes, with post-treatment increases in IFN-γ, IL-2, and IL-6, indicating activation of pro-inflammatory and T-cell pathways. However, several clinical factors such as gender, oncogene addiction and oligoprogressive disease were linked to higher IL-2/TNF-α levels, lower IL-6 and attenuation of cytokine variations, respectively. Systemic therapies also shaped immune dynamics: TKIs were associated with lower baseline cytokine levels despite positive delta variations, while ICIs correlated with higher IL-2 and IL-6 and greater post-treatment IFN-γ increases. These findings suggest that SABR triggers measurable systemic immune activation, which is further modulated by gender, tumor biology, disease burden, and concomitant therapies. Integrating cytokine profiling may help optimize patient selection and inform rational combinations of SABR with targeted and immune-based treatments.

This work fits within the scope of precision medicine, which aims to identify biomarkers that can predict therapeutic response or patient survival, taking into account that future efforts should focus on validating these findings in larger cohorts and exploring the underlying mechanisms to optimize therapeutic strategies for NSCLC patients.

To build upon these preliminary findings, future research should prioritize larger and more homogeneous cohorts to validate our prognostic models in independent patient populations. Moreover, integrating immune cell phenotyping or functional assays with soluble biomarker data would provide a more comprehensive understanding of the immune landscape. Future studies would also benefit from predefined, high-frequency sampling timepoints to more accurately capture cytokine kinetics and minimize the statistical impact of multiple testing.

## 6. Study Limits

Beyond the small sample size, several other factors may have influenced our results. First, the heterogeneity of systemic treatments (e.g., TKIs, immunotherapy) represents a significant confounder, as these agents independently modulate cytokine levels. Second, because of the exploratory nature of this pilot study involving multiple cytokine markers, there is an inherent risk of multiple testing errors; consequently, the *p*-values reported should be considered descriptive. Finally, the lack of complementary immune profiling (such as peripheral blood mononuclear cell phenotyping) and the absence of an independent validation cohort limit our ability to correlate soluble biomarkers with cellular immune dynamics.

## Figures and Tables

**Table 1:** Distribution of the study population.

Patient and Disease Characteristics		
Gender		
Male	6	31.60%
Female	13	68.40%
Age		
Median		68
Range		54–74
Histology		
Adenocarcinoma	16	84.20%
Squamous cell carcinoma	2	10.50%
Other	1	5.30%
Type of oligometastasis		
Oligoprogressive	11	57.90%
Oligometastatic	8	42.10%
EGFR		
Positive	5	26.30%
Negative	12	63.20%
Not applicable	2	10.50%
ALK		
Positive	1	5.30%
Negative	16	84.20%
Not applicable	2	10.50%
MET		
Negative	12	63.20%
Not applicable	7	36.80%
RET		
Positive	2	10.50%
Negative	11	57.90%
Not applicable	6	31.60%
BRAF V600E		
Positive	1	5.30%
Negative	15	78.90%
Not applicable	3	15.80%
KRAS		
Positive	4	21.10%
Negative	11	57.90%
Not applicable	4	21.10%
ROS1		
Negative	15	78.90%
Not applicable	4	21.10%
NTRK		
Negative	6	31.60%
Not applicable	13	68.40%
PD-L1		
<1%	4	21.10%
1 ≤ x < 50	8	42.1
≥50%	7	36.8
Radiotherapy characteristics		
SRT total dose		
Median		45 Gy
Range		21–60 Gy
Dose/Fraction		
Median		11 Gy
Range		8–24 Gy
Location of treated lesions		
Brain	5	25%
Lung	7	35%
Bone	3	15%
Liver	2	10%
Adrenal gland	3	15%
Previous Systemic Treatment		
Chemotherapy		
YES	10	52.60%
NO	9	47.40%
Immunotherapy		
YES	7	36.80%
NO	12	63.80%
Previous immunotherapy molecules		
Pembrolizumab	5	71.4%
Durvalumab	1	14.3%
Nivolumab	1	14.3%
TKI		
YES	4	21.10%
NO	15	78.90%
Previous TKI molecules		
Osimertinib	4	100%
Concurrent Systemic Treatment		
SBRT alone	7	37%
Chemotherapy		
YES	0	0%
NO	19	100%
Immunotherapy		
YES	6	31.60%
NO	13	68.4%
Concurrent immunotherapy molecules		
Pembrolizumab	4	66.6%
Atezolizumab	1	16.7%
Nivolumab	1	16.7%
TKI		
YES	6	31.6%
NO	13	68.4%
Concurrent TKI molecules		
Alectinib	1	16.66%
Lazertinib	1	16.66%
Afatinib	1	16.66%
Osimertinib	3	50%

## Data Availability

The original contributions presented in this study are included in the article. Further inquiries can be directed to the corresponding author.

## Abbreviations

The following abbreviations are used in this manuscript:

SRT	Stereotactic radiotherapy
NSCLC	Non-small-cell lung cancer
OS	Overall survival
PFS	Progression-free survival
MHC-1	Major histocompatibility complex class 1
APCs	Antigen-presenting cells
IL	Interleukin
IFN	Interferon
